# Diuretic resistance in cardiorenal syndrome: mechanisms, monitoring and phenotype-tailored management

**DOI:** 10.3389/fcvm.2025.1731305

**Published:** 2026-01-05

**Authors:** Georgios Aletras, Maria Bachlitzanaki, Maria Stratinaki, Emmanuel Foukarakis, Ioannis Petrakis, Yannis Pantazis, Michalis Hamilos, Kostas Stylianou

**Affiliations:** 1 Department of Cardiology, Venizelio General Hospital of Heraklion, Heraklion, Greece; 2 School of Medicine, University of Crete, Heraklion, Greece; 3 Second Department of Internal Medicine, Venizelio General Hospital of Heraklion, Heraklion, Greece; 4 Department of Nephrology, University General Hospital of Heraklion, Heraklion, Greece; 5 Institution of Applied and Computational Mathematics, Foundation of Research and Technology-Hellas, Heraklion, Greece; 6 Department of Cardiology, University General Hospital of Heraklion, Heraklion, Greece

**Keywords:** cardiorenal syndrome, chronic kidney disease, diuretic resistance, frailty, loop diuretics, obesity, right heart failure, urinary sodium

## Abstract

Congestion drives most hospitalizations for acute and chronic heart failure (HF), reflecting the pivotal role of sodium and water retention in disease progression. Loop diuretics remain the first-line decongestive therapy, yet up to one-third of patients exhibit an inadequate natriuretic response—defined as diuretic resistance (DR)—which is strongly associated with prolonged hospitalization, readmissions and adverse outcomes. DR is a multifactorial phenomenon arising from pharmacokinetic limitations, tubular adaptations, neurohormonal activation, and hemodynamic disturbances. Impaired renal perfusion, elevated venous pressures, and chloride depletion are key contributors that mutually reinforce one another and blunt diuretic efficacy. Early recognition through urinary sodium measurement and urine output monitoring is essential to guide therapy before resistance becomes entrenched. Beyond optimizing loop diuretic delivery, management strategies should include sequential nephron blockade, correction of electrolyte and acid–base imbalances and avoidance of excessive sodium restriction. Certain patient phenotypes—right heart failure (RHF), advanced chronic kidney disease (CKD), obesity-related HF with preserved ejection fraction (HFpEF), and frail or elderly patients—pose additional challenges due to overlapping mechanisms of resistance and increased treatment vulnerability. Each requires a tailored approach that balances decongestion with preservation of renal function and systemic perfusion. In refractory cases, extracorporeal fluid removal or peritoneal dialysis may be necessary, while newer pharmacologic agents—such as sodium–glucose cotransporter 2 inhibitors (SGLT2i), mineralocorticoid receptor antagonists (MRAs), and glucagon-like peptide-1 receptor agonists GLP-1 RAs)—offer complementary benefits. This review synthesizes mechanistic insights, bedside monitoring tools and phenotype-specific strategies for the management of DR in cardiorenal syndrome (CRS).

## 1 Introduction

Heart failure (HF) is a major public health problem, affecting more than 6 million people in the United States alone, with nearly 670,000 new cases diagnosed each year. In developed countries, the prevalence is 1%–2% of the adult population, rising steeply with age to more than 10% among individuals over 70 years. HF is the leading cause of hospitalization in patients over 65 years, and recurrent admissions for acute decompensation are associated with excess mortality and impaired quality of life. In contemporary cohorts, the vast majority of acute decompensations are driven by congestion due to sodium and water retention (1, 2).

Given the central role of congestion, diuretic therapy remains the cornerstone of symptom relief in acute and chronic HF. Loop diuretics in particular are indispensable for achieving decongestion. Their efficacy is determined by their site of action along the nephron, as all diuretics (except for osmotic agents) act by inhibiting tubular sodium transporters (Table 1) (3, 4). Yet, despite aggressive therapy, up to 20%–30% of patients fail to achieve adequate natriuresis and decongestion—a phenomenon termed diuretic resistance (DR).

**Table 1:** Classification of diuretics according to their primary site of action along the nephron and their main mechanisms.

Diuretic class	Primary site of action	Mechanism of action
Carbonic anhydrase inhibitors (e.g., Acetazolamide)	Proximal convoluted tubule	Inhibition of carbonic anhydrase
Osmotic diuretics (e.g., Mannitol)	Proximal convoluted tubule and thick ascending limb of Henle's loop	Osmotic action
Loop diuretics (Furosemide, Torasemide, Bumetanide)	Thick ascending limb of Henle's loop	Inhibition of Na^+^-K^+^-2Cl^−^ cotransporter
Thiazides (e.g., Hydrochlorothiazide) and Thiazide-like diuretics (Metolazone, Chlorthalidone, Indapamide)	Distal convoluted tubule	Inhibition of Na^+^-Cl^−^ cotransporter
Potassium-sparing diuretics (Amiloride, Triamterene)	Cortical collecting duct	Inhibition of epithelial sodium channels
Mineralocorticoid receptor antagonists (Spironolactone, Eplerenone)	Distal convoluted tubule Cortical collecting duct	Aldosterone receptor antagonism

Clinically, DR is defined as the inability to achieve negative sodium and fluid balance despite appropriate dosing (5–7). Although various definitions of DR have been proposed, no single universal standard exists (8–10). Early identification of suboptimal diuretic response is crucial, and contemporary guidelines emphasize dynamic reassessment of natriuretic response, urine output, and hemodynamic status to prevent progression toward refractory DR (1, 7, 11–13).

Importantly, what is often interpreted as DR may represent insufficient drug delivery or underdosing rather than true pharmacologic resistance. Distinguishing between these possibilities requires structured, physiologic assessment rather than empiric escalation alone. DR is consistently associated with persistent congestion, prolonged hospitalization, recurrent admissions, and poor prognosis (4, 14–16). Given its multifactorial pathophysiology—including impaired drug absorption, reduced renal perfusion, tubular adaptation, neurohormonal activation, and chloride depletion—management requires an integrated, systematic approach (7, 11, 14).

In this narrative review, we synthesize current evidence on the mechanisms, diagnostic tools, and therapeutic strategies for DR, with a dedicated emphasis on challenging clinical phenotypes—right HF (RHF), advanced chronic kidney disease (CKD), obesity-related HF with preserved ejection fraction (HFpEF), and elderly or frail patients. We explicitly present this phenotype-based framework as a conceptual clinical model intended to support bedside decision-making, rather than a validated classification system.

## 2 Literature review strategy

A comprehensive literature search was conducted in PubMed/MEDLINE**,** Embase, and Scopus from studies published between January 2000 to July 2025. The search combined controlled vocabulary (MeSH/Emtree terms) and free-text keywords using Boolean operators such as *AND*, *OR*, and *NOT*. Core concepts included “heart failure” OR “cardiorenal syndrome” AND “diuretic resistance,” “loop diuretics,” or “natriuresis,” together with mechanism- or therapy-related terms such as “chloride depletion,” “ultrafiltration,” “SGLT2 inhibitors,” “acetazolamide,” “GLP-1 receptor agonists,” “right heart failure,” “obesity,” “frailty,” and “chronic kidney disease.” Searches were restricted to English-language publications and, where applicable, to human studies.

To ensure completeness, we also manually screened reference lists from major guideline documents (ESC, AHA/ACC/HFSA, KDIGO), pivotal clinical trials, meta-analyses, and key mechanistic papers. We included randomized controlled trials, observational cohorts, mechanistic studies, and narrative or systematic reviews relevant to the pathophysiology, diagnosis, and management of DR in HF and cardiorenal syndrome.

In total, approximately 140 articles were reviewed, and nearly 100 were ultimately included based on relevance to the mechanistic, diagnostic, and phenotype-specific themes synthesized in this manuscript.

## 3 Pathophysiological mechanisms of diuretic resistance

DR is multifactorial, involving pharmacokinetic, tubular, and hemodynamic determinants. It can be broadly classified into short-term resistance, when the effect of a loop diuretic wanes after the first dose, and long-term resistance, associated with chronic administration and enhanced sodium reabsorption in the distal nephron (2, 17). These mechanisms often coexist in patients with HF and cardiorenal syndrome (CRS), forming a self-reinforcing cycle of sodium retention and impaired decongestion.

**Figure 1:**
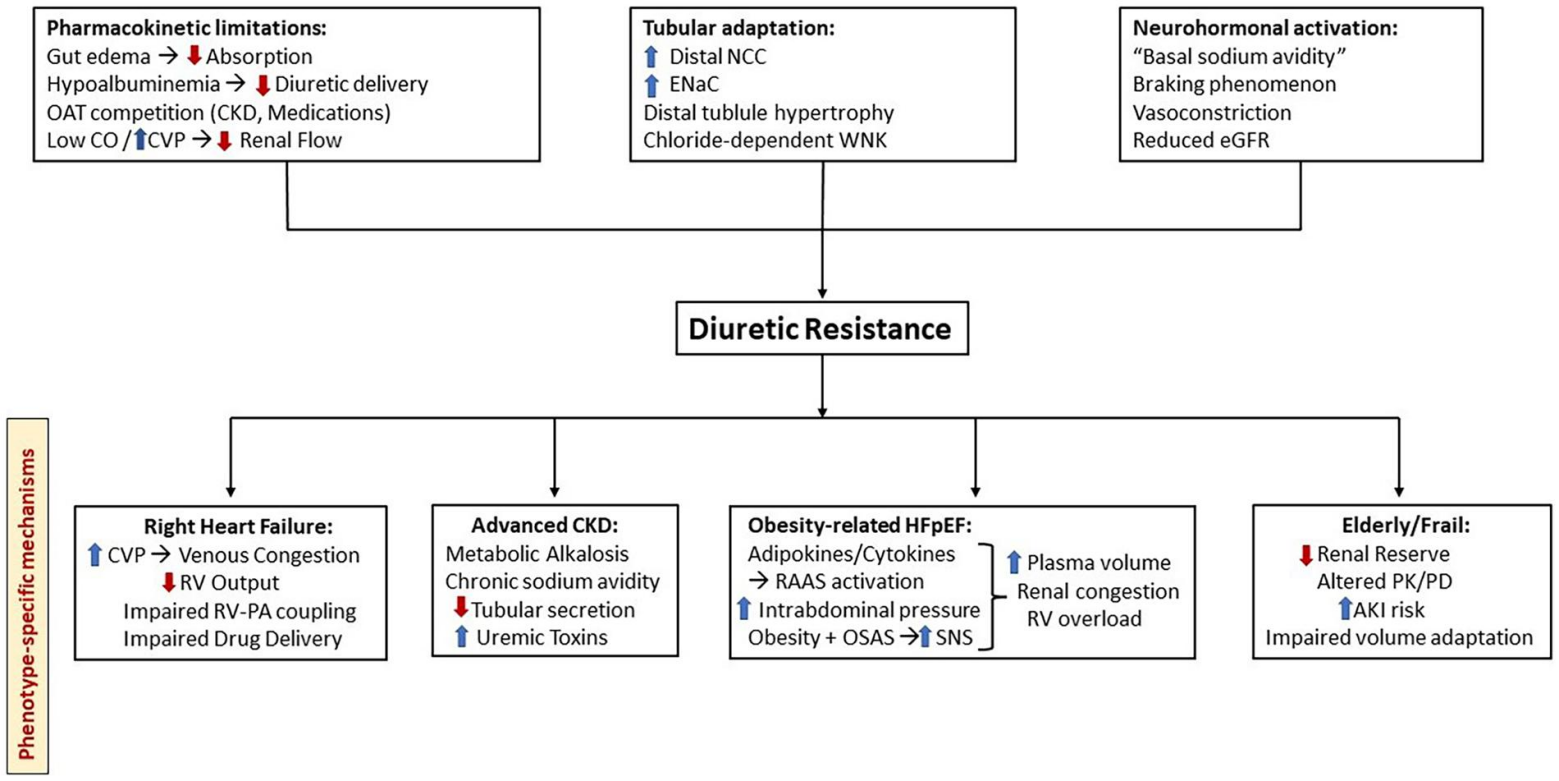
Mechanistic pathways leading to diuretic resistance and phenotype-specific amplifiers. Diuretic resistance results from three major mechanisms: pharmacokinetic limitations (impaired absorption, reduced tubular delivery, low CO, elevated CVP), tubular adaptation (upregulated NCC/ENaC, distal hypertrophy, WNK activation), and neurohormonal activation (RAAS, SNS, vasoconstriction, “basal sodium avidity”). These pathways are further intensified by specific clinical phenotypes—right heart failure, advanced CKD, obesity-related HFpEF, and frailty/older age—each contributing distinct hemodynamic, renal, or metabolic factors that blunt natriuretic response and sustain congestion. AKI, acute kidney injury; CKD, chronic kidney disease; CO, cardiac output; CVP, central venous pressure; ENaC, epithelial sodium channel; HFpEF, heart failure with preserved ejection fraction; NCC, sodium–chloride cotransporter; OAT, organic anion transporter; OSAS, obstructive sleep apnea syndrome; PK/PD, pharmacokinetics/pharmacodynamics; RAAS, renin–angiotensin–aldosterone system; RV, right ventricle; SNS, sympathetic nervous system; WNK, With-no-lysine kinase.

A schematic overview of these interacting pathways is provided in Figure 1.

### 3.1 Proximal mechanisms and drug delivery

In acute decompensated heart failure (ADHF), intestinal wall edema is common and markedly reduces the oral bioavailability of loop diuretics—particularly furosemide—by impairing absorption and delaying onset of action. For this reason, intravenous administration is preferred during the acute phase to ensure predictable and adequate natriuretic exposure until gut perfusion and absorption normalize (18, 19).

Loop diuretics such as furosemide are highly protein bound and rely on active secretion in the proximal tubule to reach their site of action. Hypoalbuminemia can reduce effective delivery by decreasing protein binding and increasing distribution volume (7, 20). Competition for tubular secretion from uremic toxins, metabolic acidosis in chronic kidney disease (CKD), or concomitant drugs may further limit excretion. Among these, nonsteroidal anti-inflammatory drugs (NSAIDs) are particularly relevant, as they not only compete for secretion but also reduce renal blood flow and blunt prostaglandin-mediated vasodilation.

In advanced HF, additional hemodynamic factors—low cardiac output (CO) and elevated central venous pressure (CVP)—further compromise renal perfusion and drug delivery. The combination of reduced renal blood flow and impaired proximal secretion directly limits the amount of loop diuretic reaching the thick ascending limb, thereby reducing pharmacologic efficacy (7, 9, 21).

### 3.2 Distal tubular adaptations

Even when drug delivery is adequate, diuretic efficacy is often blunted by compensatory sodium reabsorption downstream. Chronic exposure to loop diuretics induces structural and functional changes in the distal nephron, including hypertrophy and hyperplasia of epithelial cells, upregulation of sodium-chloride cotransporters, and activation of the epithelial sodium channel (ENaC). In the nephrotic syndrome, urinary plasmin directly activates ENaC, explaining the efficacy of amiloride in resistant edema (11, 22).

These compensatory mechanisms contribute to the phenomenon of “long-term braking,” in which the distal nephron increases sodium avidity regardless of loop diuretic dosing, thereby shifting the dose–response relationship downward and to the right (7, 9, 11).

### 3.3 Neurohormonal activation and braking phenomenon

Loss of sodium and water after loop diuretic administration stimulates counter-regulatory activation of the renin–angiotensin–aldosterone system (RAAS) and the sympathetic nervous system (SNS). This response, often referred to as the “braking phenomenon,” enhances proximal sodium reabsorption, reduces glomerular filtration, and blunts the natriuretic effect. Clinically, this is reflected by a shift of the dose–response curve downward and to the right, meaning progressively higher doses are needed to achieve the same effect, until a “ceiling dose” is reached beyond which further escalation is ineffective (9, 11).

While the braking phenomenon is well established in experimental and healthy volunteer studies, its role in ADHF appears to be less prominent. In the Mechanisms of Diuretic Resistance cohort, patients receiving intravenous loop diuretics showed increased sodium excretion not only in the first 6 h after dosing but also at 18 h—without evidence of the expected “post-diuretic” sodium retention. Interestingly, those who had a greater initial natriuretic response tended to maintain higher sodium excretion over time.

These observations suggest that in acute HF (AHF), resistance is less about an immediate counter-regulatory “braking” effect and more about a chronically heightened sodium-retaining state of the kidneys—often termed “basal sodium avidity”. This reflects long-standing neurohormonal activation (RAAS, sympathetic stimulation, vasopressin release) and renal adaptation to volume overload, which together reinforce a sodium-retentive state within the nephron. Consequently, even when loop diuretic delivery is adequate, these patients may exhibit a blunted natriuretic response because the kidney is functionally “primed” to conserve sodium rather than excrete it (7, 20, 21).

### 3.4 Role of chloride in diuretic resistance

Although sodium has traditionally been the focus in HF, recent evidence highlights chloride as a key determinant of diuretic response and prognosis. Hypochloremia, more than hyponatremia, is consistently associated with adverse outcomes and reduced diuretic efficiency. In both chronic and AHF cohorts, lower serum chloride correlates with impaired natriuresis despite adequate loop diuretic dosing, and *post hoc* analyses of the Renal Optimization Strategies Evaluation (ROSE) trial confirmed its strong prognostic value (23, 24).

Mechanistically, chloride regulates renal salt handling through its interaction with the With-no-Lysine kinases (WNK), intracellular sensors that control sodium transporters along the nephron. Low intracellular chloride activates these kinases, upregulating sodium–chloride reabsorption and counteracting the effects of loop and thiazide diuretics. Loop diuretics themselves can worsen hypochloremia, creating a vicious cycle of falling chloride, persistent neurohormonal activation, and declining therapeutic efficacy (24–27).

In addition, chloride depletion contributes to the development of metabolic alkalosis, historically termed “contraction alkalosis” but more accurately described as chloride-depletion alkalosis. In the absence of adequate luminal chloride, pendrin-mediated chloride–bicarbonate exchange in the collecting duct is impaired, perpetuating alkalemia and further reducing natriuretic responsiveness. Together, these mechanisms highlight chloride as both a mediator of resistance and a potential therapeutic target in HF (24, 26, 28).

## 4 Diagnostic and monitoring tools

Assessment of DR requires timely identification of an inadequate natriuretic response and early therapy adaptation. While weight and net fluid balance remain widely used, they correlate poorly with true sodium loss and may be misleading due to fluid redistribution rather than absolute changes in volume status (3, 29).

Since the therapeutic goal of decongestion is fundamentally sodium removal, early measurement of urinary sodium (UNa) provides the most direct and guideline-supported assessment of loop diuretic effectiveness. A spot urine sample obtained 1–2 h after intravenous loop diuretic administration correlates well with cumulative natriuresis over six hours. UNa values <50–70 mmol/L indicate an inadequate response and should prompt loop diuretic dose escalation or the addition of sequential nephron blockade (1). It is important to recognize that UNa may decline with repeated dosing even when urine output remains high, reflecting dynamic changes in renal sodium handling and neurohormonal activation (9).

Because urine concentration varies with hydration status, diuretic timing, and concomitant therapies, interpreting UNa alone can sometimes be misleading. To improve interpretability, the urinary sodium-to-creatinine (UNa/UCr) ratio adjusts sodium concentration for urine dilution. This ratio provides a more reliable assessment of natriuretic response across patients and may be particularly useful in settings of non-osmotic vasopressin activation or when agents such as sodium glucose co-transporter-2 inhibitors (SGLT2i) increase urine output while lowering sodium concentration. However, standardized cut-off values have not yet been validated, limiting its widespread clinical incorporation (30, 31).

Urine output monitoring is a simple bedside tool available in all clinical settings. A satisfactory response is generally defined as >100–150 mL/h in the first 6 h of intravenous therapy, whereas persistently low outputs despite escalating doses indicate poor diuretic responsiveness (1, 3, 12).

Contemporary European Society of Cardiology (ESC) guidelines recommend that loop diuretics in ADHF be initiated intravenously at 20–40 mg of furosemide in diuretic-naïve patients. For patients already receiving chronic outpatient loop diuretics, the recommended starting intravenous (IV) dose is approximately 2.5 times their home oral furosemide dose. Importantly, a single daily bolus of furosemide is ineffective, as post-diuretic sodium retention rapidly reverses natriuresis once drug levels fall below threshold. Therefore, initial therapy should include either two to three IV boluses within the first 24 h or a continuous infusion preceded by a loading bolus to ensure adequate tubular drug exposure and minimize diuretic-free periods. When UNa or urine output targets are not met, clinicians should escalate the loop dose, shorten dosing intervals, or transition to continuous infusion (1, 12, 20).

Beyond laboratory and clinical monitoring, point-of-care ultrasound (POCUS) provides complementary insights into congestion. Lung ultrasound detects B-lines as a sensitive and specific marker of interstitial edema, while the Venous Excess Ultrasound (VExUS) score integrates Doppler patterns from the inferior vena cava (IVC), hepatic, portal, and intrarenal veins to quantify systemic venous congestion. High VExUS scores in CRS strongly suggest impaired decongestion and support more aggressive therapeutic strategies (32–35).

Taken together, urinary biomarkers (UNa and UNa/UCr), urine output, and bedside ultrasound allow clinicians to move beyond crude measures such as weight and clinical examination, enabling a structured and physiology-guided assessment of DR. Each modality carries inherent limitations—including timing, sampling variability, operator dependence, and the influence of concomitant therapies—but their combined use markedly improves early recognition and personalized management (Table 2).

**Table 2:** Key practical indicators of diuretic response.

Indicator	Definition and clinical interpretation
Urinary sodium (UNa)	Spot urine Na^+^ 2–6 h after loop diuretic; <50–70 mmol/L indicates inadequate response and need for escalation or combination therapy
Urine output (UO)	UO >100–150 mL/hour during the first 6 h of intravenous (IV) therapy suggests satisfactory response; persistently low output signals poor responsiveness
Lung ultrasound	B-lines detect pulmonary congestion
Venous Excess Ultrasound (VExUS)	Quantification of systemic venous congestion, guiding aggressive decongestion when score is high

IV, Intravenous; Una, Urinary sodium; UO, Urine output.

## 5 Challenging clinical scenarios in diuretic resistance and CRS

To operationalize a phenotype-guided approach to DR, this review focuses on four clinical profiles frequently encountered in CRS—right-sided HF, advanced CKD, obesity-related HF, and frailty. These phenotypes share common mechanisms of sodium retention yet differ in their dominant pathophysiologic drivers, diagnostic challenges, and therapeutic vulnerabilities, enabling a more individualized and mechanistically informed treatment strategy. These phenotypes are especially challenging to treat, while these groups are underrepresented in clinical trials but commonly seen in clinical settings. Each phenotype exhibit distinct mechanisms that reduce diuretic response and require a personalized treatment approach.

### 5.1 Right heart failure

RHF is characterized by the inability of the right ventricle to maintain adequate forward flow, resulting in systemic venous hypertension and congestion. In this phenotype, DR is particularly common and reflects a complex interplay of impaired renal perfusion, chronic loop diuretic exposure, venous hypertension, low cardiac output, hypotension, and acute kidney injury (36).

Elevated right-sided filling pressures compromise renal prefusion by increasing renal venous pressure, reducing the transrenal perfusion gradient and promoting renal and intestinal congestion that limits loop diuretic delivery. In advanced stages, impaired right-to-left ventricular coupling can cause left-sided underfilling and ultimately cardiogenic shock. Congestion is the predominant clinical feature and a principal driver of renal deterioration and multiorgan dysfunction (37–39). Hemodynamically, these patients exhibit marked preload dependence and afterload sensitivity of the right ventricle (RV). Adequate preload is necessary to preserve stroke volume through the Frank–Starling mechanism; however, excessive filling pressures rapidly translate into systemic venous hypertension, hepatic congestion, and renal stasis. Conversely, over-aggressive decongestion or ultrafiltration can lower RV output and LV filling, leading to systemic hypotension and renal hypoperfusion. Increases in pulmonary vascular resistance—due to hypoxia, acidosis, or vasoconstrictor use—further elevate RV afterload and impair forward flow. This delicate balance between maintaining adequate preload and avoiding excessive afterload defines the therapeutic challenge in managing decompensated RHF (37, 38, 40, 41).

The primary goal is to relieve congestion since elevated central venous pressure (CVP) remains a key finding and a major factor in CRS (42, 43). Loop diuretics are the cornerstone of treatment, but higher doses are often necessary, and using sequential nephron blockade with thiazides, metolazone, or acetazolamide may be beneficial (36, 40, 41). SGLT2i can also offer additional natriuresis and kidney protection, without significant hemodynamic effects (44). Decongestion should be prioritized even in the presence of borderline blood pressure, provided there is no evidence of hypoperfusion—such as cool extremities, rising lactate, altered mental status or a narrow pulse pressure. In such cases, inotropes/vasopressors can be used to maintain systemic perfusion and enable effective diuresis; these agents support decongestion but should not replace it (1, 3, 36, 40).

Because patients with RHF are highly preload dependent, therapy should aim to reduce venous congestion while preserving right ventricular preload to maintain forward flow. Excessive volume removal may precipitate hypotension, reduced cardiac output and “true” worsening renal function (WRF). Continuous monitoring of volume status, urine sodium and hemodynamics is essential to tailor therapy and prevent these complications (36, 38).

When impaired contractility is a major contributor, inotropic support can improve right ventricle–pulmonary artery coupling. The choice of agent depends on blood pressure, concomitant therapies, and renal function (36).

Dobutamine remains the most commonly used first-line inotrope. It has a rapid onset, a short half-life, and modest vasodilatory properties, making it suitable for patients with RV dysfunction and borderline hemodynamics. However, it can provoke tachyarrhythmias at higher doses and tolerance may develop during prolonged infusions. Milrinone, by contrast, exerts stronger pulmonary and systemic vasodilation, leading to greater reductions in filling pressures. It is often preferred in patients receiving chronic *β*-blocker therapy, as its mechanism bypasses adrenergic signaling. Tachycardia is less frequent than dobutamine, and tolerance is uncommon. On the downside, milrinone carries a higher risk of hypotension—especially with bolus dosing—owing to its long half-life and vasodilator effects. In such cases, concurrent vasopressor support may be necessary. Because it is partly renally cleared, dosing must be adjusted for kidney function (36, 40, 45). When pulmonary vasoconstriction predominates, therapy should prioritize afterload reduction—optimizing oxygenation and ventilation, correcting acidosis, and avoiding pure *α*-agonists that raise pulmonary vascular resistance (46, 47).

Levosimendan, a calcium sensitizer with additional vasodilatory effects, offers an attractive alternative to conventional adrenergic inotropes. Unlike catecholamines, it enhances contractility without increasing intracellular calcium or oxygen demand. In clinical studies, it has been associated with improved RV systolic performance, reduced pulmonary artery pressures, and greater diuretic responsiveness. Observational data suggest a neutral safety profile regarding mortality, in contrast to the signal of harm seen with chronic adrenergic inotrope use. Levosimendan can be used alone, but in hypotensive patients it is often combined with norepinephrine to maintain perfusion pressure. Some centers also employ it prophylactically in patients at high risk of RV dysfunction after left ventricular assist device (LVAD) implantation (48–50).

When hypotension dominates (mean arterial pressure <65 mmHg or systolic blood pressure <80–90 mmHg with hypoperfusion), the focus shifts to vasopressors. Norepinephrine is generally preferred, as it restores systemic pressure with limited chronotropic stimulation and can improve the pulmonary/systemic vascular resistance ratio. Vasopressin may be added when catecholamine-sparing support is desired, as it augments mean arterial pressure without substantially worsening pulmonary pressures. Epinephrine can be considered in refractory cases, while pure *α*-agonists such as phenylephrine are usually avoided because they increase RV afterload. In practice, contractile failure and venous congestion often coexist. Decongestion should continue even in patients requiring inotropes or vasopressors, with hemodynamic support serving to maintain perfusion and allow effective volume removal, since relief of venous hypertension often restores renal perfusion and may even lead to improvement in systemic blood pressure (51, 52).

Continuous monitoring includes rhythm surveillance, urine output measurement, serial UNa assessment, and lactate measurements every 4–6 h in shock states, together with bedside echocardiographic assessment of filling pressures and RV function. Therapy should be reassessed frequently—typically every 6–12 h—to guide de-escalation. Once mean arterial pressure (MAP) is stabilized and effective diuresis achieved, adrenergic agents should be tapered first, as minimizing their duration reduces arrhythmic and ischemic risks (Table 3) (12, 36, 40, 53).

**Table 3:** Inotropes and vasopressors in right heart failure: Key considerations.

Agent	Main action	When to consider	Advantages	Limitations/Cautions
Dobutamine	*β*1-agonist (inotropy), mild β2 vasodilation	First-line inotrope in low output with near-normal blood pressure	Rapid onset/offset, short half-life, less hypotension	Tachyarrhythmias at higher doses; tolerance with prolonged use; limited efficacy on β-blockers
Milrinone	PDE3 inhibitor → ↑cAMP (inotropy + vasodilation)	Low output on β-blocker or high pulmonary vascular resistance	Strong pulmonary vasodilation, less tachycardia, minimal tolerance	Hypotension (long half-life); requires renal adjustment
				Vasopressor often needed to anticipate hypotension
Levosimendan	Calcium sensitiser + KATP channel opener (inotropy + vasodilation)	Alternative or adjunct when adrenergic inotropes limited; bridge before LVAD; severe RHF with congestion	Improves RV contractility, lowers PAP, enhances diuretic response, neutral mortality signal	Limited availability; hypotension risk (combine with vasopressors if needed); evidence mostly observational
Norepinephrine	*α*1 (vasoconstriction), mild β1 (inotropy)	Hypotension with RHF, especially if congestion persists	Raises systemic BP, improves perfusion, favorable PVR/SVR ratio	May increase afterload if excessive, risk of arrhythmias
Vasopressin	V1 receptor agonist (vasoconstriction), preserves renal perfusion	Catecholamine-sparing adjunct when SVR is low and PVR concerns	Maintains MAP without major rise in PVR, supports renal GFR	High doses → bradycardia, ↓RV contractility; titrate carefully

cAMP, cyclic adenosine monophosphate; GFR, glomerular filtration rate; KATP, adenosine triphosphate—sensitive potassium; LVAD, left ventricular assist device; MAP, mean arterial pressure; PAP, pulmonary arterial pressure; PDE3, phosphodiesterase-3; RHF, right heart failure; RV, right ventricle; SVR, systemic vascular resistance.

For patients who remain congested despite maximum pharmacological therapy, renal replacement strategies may be necessary. Ultrafiltration has yielded mixed results, whereas peritoneal dialysis (PD) provides gradual, hemodynamically stable fluid removal and can sometimes be continued outside the hospital setting. In cases progressing to severe contraction failure or cardiogenic shock, escalation to mechanical circulatory support (MCS)—such as extracorporeal membrane oxygenation (ECMO) or percutaneous RV assist devices—may be essential as a bridge to recovery or transplantation (40, 54).

**Figure 2:**
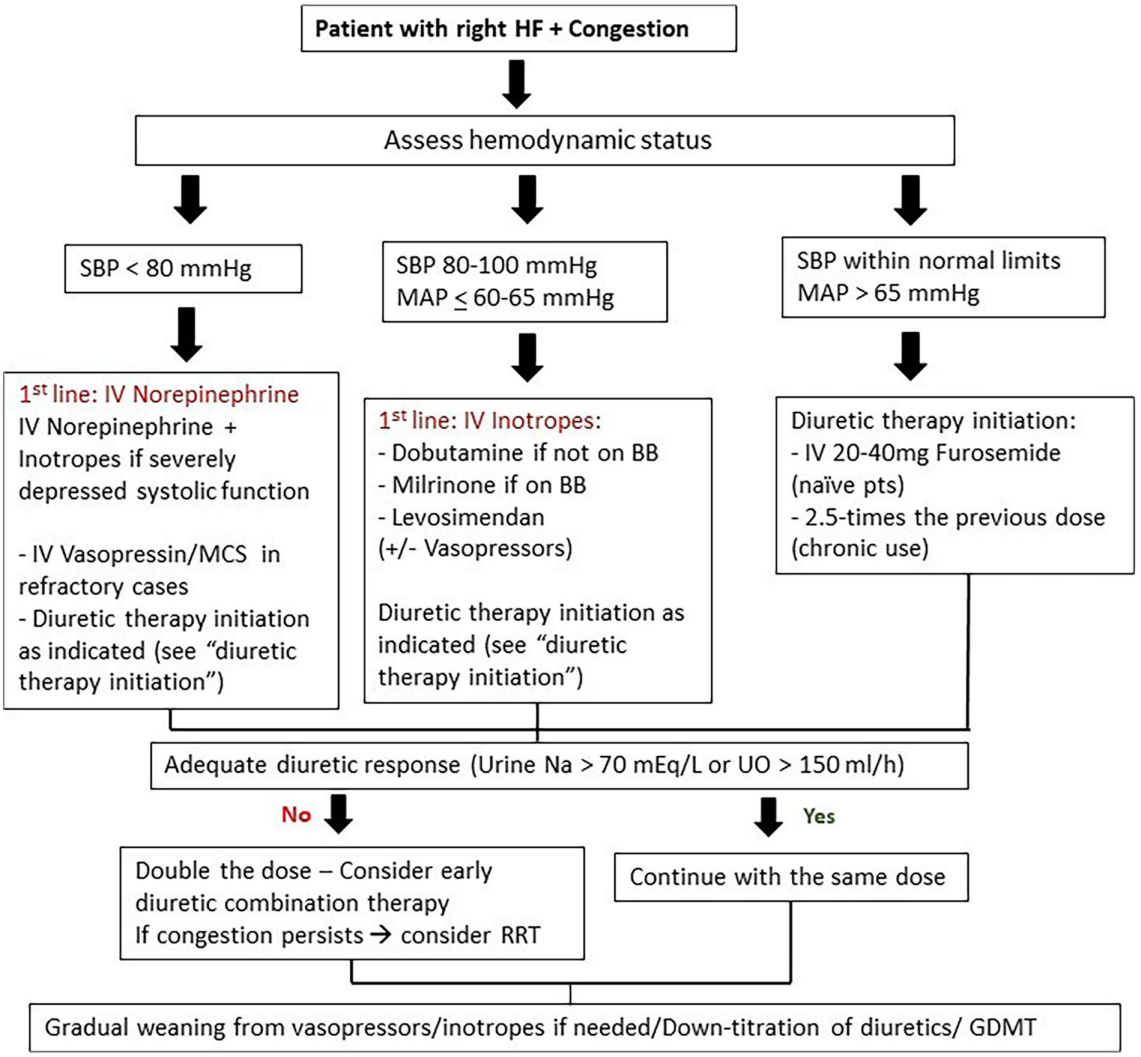
Algorithm for the management of right heart failure with diuretic resistance, highlighting the interplay between decongestion, hemodynamic stabilization, and escalation to advanced therapies. GDMT, Guideline directed medical therapy; HF, Heart Failure; IV, Intravenous; MAP, Mean Arterial Pressure; MCS, Mechanical circulatory support; SBP, Systolic Blood Pressure; UO, Urine output.

In summary, RHF is a distinct phenotype of CRS defined by systemic venous hypertension and impaired RV–pulmonary artery (PA) coupling, in which congestion is both the cause and consequence of DR. Management is not strictly sequential but often requires simultaneous decongestion and circulatory support, combined with close monitoring and escalation to advanced therapies when standard measures fail (Figure 2, Table 4) (36, 38, 40).

**Table 4:** Key therapeutic pitfalls in decompensated right heart failure. Avoiding these errors can optimize decongestion, preserve renal function, and prevent hemodynamic instability.

Pitfall	Preferred Approach/Rationale
1. Single large daily loop bolus	Split dosing or continuous infusion to minimize post-diuretic sodium retention and maintain steady natriuresis.
2. Ignoring chloride	Correct hypochloremic metabolic alkalosis; use potassium chloride when hypokalemia coexists to restore distal sodium delivery and diuretic responsiveness.
3. Halting diuresis for minor creatinine rises	Small increases often reflect hemoconcentration rather than structural injury; reassess perfusion, urine output, and congestion before de-escalation.
4. Using pure α-agonists (e.g., phenylephrine) in right ventricular failure	Avoid agents that raise pulmonary vascular resistance; prefer norepinephrine (± vasopressin) to support systemic pressure while preserving right ventricular output.
5. Under-monitoring	Monitor urine sodium, urine output, and hemodynamics (including bedside echo parameters) to guide timely dose adjustment and escalation.

### 5.2 Advanced chronic kidney disease

Patients with advanced CKD who present with ADHF represent one of the most challenging clinical scenarios in diuretic management. Congestion is not only more frequent and severe in this population, but also more tightly linked to outcomes, with lung congestion emerging as a stronger predictor of mortality than traditional risk factors such as diabetes or hypertension. This phenotype is characterized by profoundly altered pharmacokinetics, impaired tubular function, and heightened neurohormonal activation, making DR more prevalent and more difficult to reverse than in other CRS profiles (55–57).

The first step in approaching these patients is to address modifiable contributors. Excessive sodium restriction, long considered fundamental, is now being questioned, as very low salt intake has been associated with worse outcomes, hyponatremia, and hypochloremia—all of which may blunt diuretic efficacy. A moderate restriction—no more than 5 g of salt per day in patients with HF—is therefore preferable (58).

Equally important is the correction of hypochloremic alkalosis, as restoring chloride levels with potassium chloride or other chloride salts can help re-establish distal tubular sodium delivery and improve loop diuretic responsiveness. Potassium chloride remains the preferred option when hypokalemia coexists, whereas in normokalemic or hyperkalemic patients, chloride supplementation can be achieved using chloride–arginine or lysine chloride preparations. Experimental and early clinical studies have shown that chloride repletion (e.g., lysine chloride 115 mmol/day) increases serum chloride concentrations and favorably modulates neurohormonal and volume-related parameters, suggesting that hypochloremia in HF is not merely a marker of disease severity but a potentially modifiable therapeutic target (27, 59).

Potassium disturbances should also be addressed, and new potassium binders such as patiromer or sodium zirconium cyclosilicate allow the safe continuation of renin–angiotensin–aldosterone system inhibitors, which remain beneficial in HF and CKD despite concerns about hyperkalemia (55, 56, 60, 61).

Loop diuretics remain the cornerstone of treatment, but higher doses are often required due to impaired tubular delivery and competition at organic anion transporters. Strategies to optimize loop therapy include splitting the daily dose to reduce drug-free intervals, switching to agents with more predictable bioavailability (torsemide or bumetanide over furosemide), and using the intravenous route in decompensated patients or in the presence of gut edema. Continuous infusion may provide more stable exposure and limit rebound sodium retention, but randomized data have not demonstrated clear superiority over intermittent bolus administration, so the choice should be individualized (11, 55, 56, 60).

When congestion persists, sequential nephron blockade is the most effective pharmacological strategy. Recent evidence helps refine this approach. In the Acetazolamide in Acute Decompensated Heart Failure with Volume Overload (ADVOR) trial, acetazolamide added to loop diuretics significantly improved early decongestion, particularly in patients with higher baseline bicarbonate levels, but its effect was attenuated among those chronically exposed to high loop diuretic doses. Conversely, in the CLOROTIC trial, thiazide-like diuretics showed greater efficacy in patients receiving higher baseline loop doses, suggesting that chronic loop exposure induces distal tubular adaptation that limits the impact of proximal blockade. Therefore, acetazolamide appears most useful in patients with limited prior diuretic exposure or metabolic alkalosis, whereas thiazide-type agents may be preferable in those already on high-dose chronic loop therapy (3, 10, 56, 62–67).

Beyond natriuresis, acetazolamide may also blunt neurohormonal activation by reducing proximal sodium reabsorption, particularly in patients not treated with renin–angiotensin system inhibitors. In ADVOR, patients with metabolic alkalosis (serum bicarbonate ≥27 mmol/L) experienced higher diuretic and natriuretic responses and shorter hospital stays compared with those with normal bicarbonate levels. These findings support early acetazolamide use to prevent loop diuretic–induced metabolic alkalosis and mitigate secondary resistance. However, its role in eGFR <20 mL/min/1.73 m^2^ remains uncertain, as such patients were excluded from pivotal trials (62, 64, 68, 69).

Thiazide-based regimens, as demonstrated in the CLOROTIC study were associated with higher rates of hypokalemia, consistent with the expected physiological consequences of distal sodium delivery and aldosterone-mediated potassium loss. Neither ADVOR nor CLOROTIC demonstrated differences in readmission or mortality, suggesting that while sequential nephron blockade enhances short-term decongestion, it does not translate into long-term prognostic benefit. This highlights the importance of structured outpatient follow-up and specialized cardiorenal programs to sustain euvolemia and optimize chronic therapy once congestion is resolved (64, 65, 69, 70).

Mineralocorticoid receptor antagonists (MRAs) or epithelial sodium channel blockers may be considered in selected cases, particularly when hypokalemia or metabolic alkalosis complicate treatment, though the risk of hyperkalemia in CKD requires vigilance. SGLT2i also play a role; although their natriuretic effect diminishes at low estimated glomerular filtration rates, they remain safe to initiate above guideline thresholds and provide long-term renal and cardiovascular protection (55, 56, 71–74).

Adjunct strategies have been proposed for truly refractory cases. Combining hypertonic saline with loop diuretics can acutely enhance natriuresis and shift the dose–response curve, particularly in patients with profound hyponatremia or hypochloremia, though results are heterogeneous and the risk of acute kidney injury with high chloride loads limits widespread adoption. Vasopressin antagonists such as tolvaptan reliably correct hyponatremia and increase free water clearance, but they have failed to improve long-term outcomes and are therefore reserved for patients with symptomatic hyponatremia. Limited data also suggest a potential role for urea as a low-cost osmotic agent to promote free water clearance and correct dilutional hyponatremia in selected patients with refractory HF. Small studies indicate that urea may improve serum sodium and facilitate decongestion without adversely affecting renal function, although evidence remains scarce and its use has not yet been validated in large randomized trials (75, 76). Modest rises in serum Cr during aggressive diuresis should not prompt premature therapy de-escalation, as they often reflect hemoconcentration rather than structural kidney injury. Preserving renal function is important, but adequate volume removal remains the cornerstone for improving symptoms and outcomes (60, 77, 78).

When pharmacologic strategies fail, extracorporeal fluid removal must be considered. Ultrafiltration provides predictable sodium and water removal, but randomized trials such as CARRESS-HF have shown worse renal outcomes and higher adverse event rates compared with stepped pharmacologic therapy. For carefully selected patients with true diuretic resistance, ultrafiltration remains an option, but its resource intensity and complication profile restrict its use. Peritoneal dialysis offers a gentler, continuous means of decongestion with fewer hemodynamic shifts, and observational studies suggest improvements in symptoms, hospitalizations, and quality of life, though robust randomized evidence is lacking. In unstable patients with concomitant acute kidney injury, continuous renal replacement therapy may be the only feasible option (55, 60, 79).

In summary, advanced CKD represents a distinct CRS phenotype in which impaired tubular handling, reduced diuretic delivery, and chronic neurohormonal activation converge to limit natriuretic response. Moderate sodium restriction, correction of electrolyte and acid–base abnormalities, optimization of loop diuretics, and sequential nephron blockade form the cornerstone of management. Adjunctive measures such as hypertonic saline or vasopressin antagonists may be considered selectively, while extracorporeal therapies should be reserved for refractory cases. The key principle is to prioritize effective decongestion while recognizing that small rises in Cr are often acceptable in the pursuit of improved symptoms and outcomes (10, 55, 60) (Table 5).

**Table 5:** Summary of pharmacologic and extracorporeal options for managing diuretic resistance in advanced chronic kidney disease.

Therapeutic agent	Indications or most suitable context	Key considerations:
Loop diuretics (Furosemide, Torsemide, Bumetanide)	First-line therapy for congestion; essential even in advanced CKD	Higher doses often required due to reduced tubular secretion; Split dosing or continuous infusion may reduce post-dose sodium retention.
Thiazide or thiazide-like diuretics (e.g., metolazone)	Add-on therapy in patients chronically exposed to high-dose loop diuretics or with persistent congestion despite loop optimization	Greater efficacy in patients on chronic loop therapy (CLOROTIC trial); monitor for hypokalemia, hyponatremia, and hypotension.
		They should be administered 30–60 min before loop diuretics to enhance sequential nephron blockade
Acetazolamide	Early adjunct in loop-naïve patients or those with metabolic alkalosis or elevated bicarbonate (≥27 mmol/L)	Enhances proximal sodium excretion and prevents metabolic alkalosis (ADVOR trial); less effective in patients on chronic high-dose loops; not tested in eGFR < 20 mL/min/1.73 m^2^.
Mineralocorticoid receptor antagonists (MRA)/ENaC blockers (e.g., Amiloride)	Selected patients with hypokalemia or metabolic alkalosis complicating therapy	Use with caution due to hyperkalemia risk in CKD; close electrolyte monitoring required.
SGLT2 inhibitors	Adjunct for long-term renal and cardiovascular protection; safe to initiate above guideline eGFR thresholds	Natriuretic effect diminishes at low eGFR but renal and CV benefits persist; may complement neurohormonal blockade.
Hypertonic saline + Loop diuretic	Consider in profound hyponatremia or hypochloremia unresponsive to standard therapy	May acutely shift the dose–response curve; results heterogeneous; avoid excessive chloride loads to prevent AKI.
Vasopressin antagonists (e.g., Tolvaptan)	Reserved for symptomatic hyponatremia refractory to other measures	Improves serum sodium and free-water clearance but no long-term mortality benefit.
Ultrafiltration/Peritoneal dialysis	Refractory congestion despite maximal pharmacologic therapy	UF may worsen renal outcomes (CARRESS-HF); PD offers gradual, hemodynamically stable decongestion and may improve quality of life.

AKI, acute kidney injury; CKD, chronic kidney disease; CVD, cardiovascular disease; eGFR, estimated glomerular filtration rate; ENaC, epithelial sodium channels; MRA, mineralocorticoid receptor antagonists; PD, peritoneal dialysis; SGLT2, sodium-glucose co-transporter 2; UF, ultrafiltration.

### 5.3 Obesity

Obesity represents a growing and particularly complex phenotype in acute and chronic HF, with implications extending from diagnosis to treatment response. In the acute setting, the evaluation of congestion is considerably more challenging than in other patient groups. Excess adiposity decreases the accuracy of both the physical examination and noninvasive imaging modalities. Traditional clinical signs—such as jugular venous distension, pulmonary rales, or peripheral edema—are often obscured, while echocardiographic indices and natriuretic peptide levels systematically underestimate the degree of circulatory congestion. As a result, hemodynamic congestion can remain clinically silent until it becomes advanced, complicating both the diagnosis and the titration of decongestive therapy. This phenotype is unique because volume overload often masks itself clinically, while neurohormonal activation and renal congestion develop early—driving DR even before overt edema or weight gain appear (80–82).

From a pathophysiological perspective, adipose tissue is not merely an inert reservoir but an active endocrine organ. It produces a variety of pro-inflammatory cytokines, adipokines, and mineralocorticoid-like factors that promote sodium retention, vascular stiffness, and sympathetic activation. Sympathetic overactivity is particularly pronounced when obesity coexists with obstructive sleep apnea, further enhancing neurohormonal activation and sodium avidity. These mechanisms contribute to plasma volume expansion, renal congestion, and right ventricular overload, particularly in patients with HFpEF (83–86). Consequently, obesity is associated with higher rates of volume overload, a greater need for diuretics, and a poorer diuretic response. The coexistence of obesity-related kidney dysfunction further compounds these effects. Increased intra-abdominal pressure reduces renal perfusion, while ectopic fat deposition within the kidney promotes inflammation, oxidative stress, and tubulointerstitial fibrosis. Together, these factors blunt natriuretic efficiency and reinforce DR (84, 87–89).

In clinical practice, patients with obesity frequently require higher doses of loop diuretics to achieve an adequate response, reflecting both altered pharmacokinetics and increased volume of distribution, while chronic high-dose loop exposure further amplifies neurohormonal activation and sodium avidity, perpetuating DR (87, 89). Weight-based dosing may partially mitigate underdosing, but clinicians must balance this with the risk of hypotension and renal dysfunction, particularly in those with comorbid CKD (1, 11, 14). When standard loop diuretic therapy fails, the same stepwise approach used in other resistant states applies: sequential nephron blockade with thiazide or thiazide-like agents, and in selected cases, the addition of acetazolamide to counteract metabolic alkalosis or proximal sodium reabsorption. SGLT2i may further enhance natriuresis and improve renal hemodynamics, although their diuretic effect is modest compared with classical agents (20, 89).

Beyond decongestion, addressing obesity itself has emerged as a cornerstone of long-term management. Weight reduction improves symptoms, exercise capacity, and overall quality of life in patients with HFpEF, while modest weight loss may also benefit those with reduced ejection fraction. Recent trials have highlighted the potential of metabolic therapies to modify both congestion and diuretic needs. In the SUMMIT trial, treatment with tirzepatide in obese HFpEF patients improved functional status and reduced estimated blood volume. Similarly, pooled analyses from the STEP-HFpEF and STEP-HFpEF-DM trials demonstrated that semaglutide not only improved HF outcomes but also decreased the requirement for loop diuretics by up to 17%, compared with an increase in the placebo group. Patients receiving semaglutide were three times more likely to have their loop diuretic dose reduced and three times less likely to require escalation. These findings position metabolic therapies not only as weight-loss agents but also as modulators of congestion oand DR through improvements in endothelial function, renal hemodynamics and inflammatory pathways (82, 87, 90).

In the chronic setting, comprehensive management of obesity should combine caloric restriction, increased physical activity, and, when indicated, pharmacologic or surgical interventions. Beyond symptomatic relief, sustained weight loss can slow the progression of renal dysfunction, reduce proteinuria, and improve glomerular filtration rate in obese patients with concomitant CKD. Bariatric surgery, when feasible, has been shown to prevent renal decline and reduce cardiovascular morbidity. The introduction of glucagon-like peptide-1 (GLP-1) and dual glucose-dependent insulinotropic peptide (GIP)/GLP-1 receptor agonists has transformed the therapeutic landscape, offering robust weight reduction with additional cardiovascular and renal protection (82, 87, 91, 92).

In summary, obesity amplifies the risk of congestion and DR through overlapping hemodynamic, neurohormonal, and renal mechanisms, while also complicating the clinical recognition of volume overload. Management requires higher or combination diuretic dosing in the acute setting and targeted weight reduction strategies in the chronic phase. The growing evidence supporting GLP-1–based therapies provides new hope for improving both symptom control and long-term outcomes in this difficult-to-treat population (Table 6) (84, 87–89).

**Table 6:** Key clinical implications in obese patients with acute or chronic heart failure presenting with diuretic resistance.

Obesity and diuretic resistance—Key clinical implications:
**Diagnostic complexity:**
Congestion is easily underestimated. Clinical signs (e.g., jugular vein distension, peripheral edema etc.) and natriuretic peptides lose sensitivity, while echocardiography underestimates filling pressures.
**Pathophysiological drivers:**
Adipose tissue acts as an endocrine organ, promoting sodium retention, plasma volume expansion and inflammation—factors that increase congestion risk and diuretic demand.
**Diuretic management:**
Higher or weight-adjusted loop diuretic doses are often required. Combination strategies (thiazide-like agents/acetazolamide) may be necessary to overcome resistance, while SGLT2i provide modest additive benefit. Whenever feasible, guideline-directed medical therapy should be initiated or up-titrated to optimize neurohormonal blockade and support decongestion.
**Chronic setting:**
Sustained weight reduction through lifestyle, pharmacologic or surgical interventions improves functional status, slows renal decline and reduces cardiovascular mortality. GLP-1 and GIP/GLP-1 receptor agonists (e.g., semaglutide, tirzepatide) improve symptoms, reduce estimated blood volume and lower loop diuretic requirements.
**Clinical priorities:**
Effective decongestion remains essential, but parallel treatment of obesity is key to breaking the cycle of congestion, renal impairment and diuretic resistance.

Bold text indicates section headings highlighting key clinical domains.

### 5.4 Elderly and frail patients: balancing decongestion and preservation

Elderly and frail HF patients represent a growing and particularly complex population, in whom diuretic resistance often develops in the context of multiple coexisting conditions and diminished physiological reserve. Frailty, a dynamic state of reduced resilience to stressors, amplifies vulnerability to volume shifts, electrolyte imbalances, and adverse drug reactions. Most older HF patients also suffer from multimorbidity—atrial fibrillation, chronic kidney disease, hypertension, dementia, or valvular disease—requiring cautious polytherapy and close interdisciplinary coordination. This phenotype is distinct because age-related changes in drug handling, neurohormonal activation, and sarcopenia reduce both diuretic delivery and tolerance to aggressive volume removal, making the balance between decongestion and preservation uniquely challenging (93, 94).

The pharmacokinetics and pharmacodynamics of diuretics are substantially altered in advanced age. Reduced renal plasma flow and tubular secretion, lower serum albumin, and changes in body composition attenuate loop diuretic delivery and potency. As a result, older adults frequently require higher or combination diuretic doses to achieve decongestion, but they are equally prone to overdiuresis, hypotension, and acute kidney injury. Clinical evaluation is further complicated by unreliable physical findings: edema may stem from hypoalbuminemia rather than congestion, and jugular venous distension or weight change may be misleading in sarcopenic individuals. Therefore, treatment must be guided by comprehensive assessment—urine output, daily weight trends, and, when available, urinary sodium or non-invasive hemodynamic monitoring (93–95).

In this setting, gradual decongestion with careful monitoring is preferred over rapid fluid removal. Thiazide-like agents or acetazolamide can be added temporarily for sequential nephron blockade, but only under close electrolyte and renal function surveillance. Guideline-directed medical therapy (GDMT) remains beneficial and should not be withheld solely due to age or frailty (93, 94). Although GDMT is often under-prescribed in advanced age, evidence indicates that use of at least one GDMT class—ACE inhibitors/ARNI, *β*-blockers, MRAs, or SGLT2i—reduces hospitalizations and mortality even in advanced age. Among these, SGLT2i appear well tolerated in the frailest categories, though vigilance for dehydration, genitourinary infections, or hypotension remains essential (93, 94, 96–99).

Ultimately, diuretic management in frail elderly patients is a delicate balancing act between decongestion and preservation. The therapeutic goal is not maximal diuresis but sustained euvolemia, maintenance of renal function, and preservation of autonomy and quality of life through individualized, closely supervised care (Table 7) (93, 94).

**Table 7:** Summary of key challenges contributing to diuretic resistance and therapeutic complexity in elderly and frail patients with heart failure.

Key challenges in elderly and frail HF patients:
Altered pharmacokinetics and reduced renal perfusion limiting diuretic efficacy
Increased risk of adverse effects due to high comorbidity burden and polypharmacy
Heightened vulnerability to volume shifts, hypotension and electrolyte disturbances
Limited reliability of congestion signs due to sarcopenia or hypoalbuminemia
Need for gradual decongestion to preserve renal hemodynamic stability
Underuse of guideline-directed therapies despite proven clinical benefit

Bold text indicates section headings highlighting key challenges.

## 6 Conclusions

DR remains a major therapeutic challenge in acute and chronic HF, driven by overlapping mechanisms involving impaired drug delivery, tubular adaptation, neurohormonal activation, venous congestion and electrolyte disturbances. Recognition of these mechanisms is essential, as DR is not a uniform entity but a heterogeneous syndrome that manifests differently across patient phenotypes.

Traditional bedside parameters such as weight change or net diuresis often fail to capture the true natriuretic response. Monitoring strategies that incorporate early urinary sodium, urine output trends, and point-of-care ultrasound markedly enhance the clinician's ability to detect inadequate response early, before resistance becomes entrenched. These approaches complement careful clinical assessment and provide actionable, real-time feedback that informs treatment escalation.

Therapeutically, loop diuretics remain the foundation of decongestion, but an individualized, physiology-guided approach is essential. Sequential nephron blockade, correction of hypochloremia or metabolic alkalosis, SGLT2 inhibition, and in selected patients acetazolamide or thiazide-type agents can substantially improve response. Adjunctive strategies—including hypertonic saline, vasopressin antagonists, or extracorporeal therapies—should be reserved for carefully selected cases after modifiable contributors have been addressed.

Importantly, a phenotype-based framework provides a useful lens for clinical decision-making. Right-sided HF, advanced CKD, obesity-related HF, and frailty each present with distinct mechanisms of DR, diagnostic limitations, and therapeutic vulnerabilities. Tailoring management to these profiles may improve both short-term decongestion and long-term outcomes. Although no single intervention overcomes all forms of DR, integrating mechanistic insights with phenotype-specific management allows for more precise and effective treatment strategies.

In summary, DR is both a marker and mediator of worse outcomes in HF. Effective management requires early identification, structured monitoring, correction of pathophysiological drivers, and an individualized therapeutic plan informed by the patient's clinical phenotype. Continued research is needed to refine phenotype-based algorithms, validate emerging biomarkers, and determine which combinations of therapies yield durable benefits in diverse HF populations.
